# Baseline type 2 biomarker levels and clinical remission predictors in children with asthma

**DOI:** 10.3389/fimmu.2025.1492644

**Published:** 2025-05-28

**Authors:** Mengmeng Chen, Congcong Li, Qiuyan Yang, Huijie Zhang, Yanli Zhang, Na Wang, Jingcheng Dong

**Affiliations:** ^1^ Department of Integrative Medicine, Huashan Hospital, Fudan University, Shanghai, China; ^2^ Shanghai Institute of Infectious Disease and Biosecurity, Fudan University, Shanghai, China; ^3^ Department of Pediatrics, Third Affiliated Hospital of Zhengzhou University, Zhengzhou, China; ^4^ Children’s Hospital of Fudan University, Shanghai, China

**Keywords:** pediatric asthma, Th2 biomarkers, thymic stromal lymphopoietin, clinical asthma remission, biomarker prediction

## Abstract

**Background:**

Few studies have investigated the relationship between baseline type 2 biomarker levels and clinical features in pediatric asthma, particularly in different asthma stages, which may inform prognosis and remission.

**Objective:**

To explore the association between baseline Th2 biomarker levels and clinical manifestations in pediatric asthma, identifying predictors of clinical remission.

**Methods:**

The study included 172 children with a mean age of 6.87 ± 3.04 years, comprising 119 asthma patients and 53 non-respiratory symptom controls. Clinical evaluations such as lung function tests, FeNO, total IgE, blood eosinophil counts, and skin tests were conducted. Serum biomarkers (TSLP, IL-4/5/13, TARC, Periostin), and IgE were measured by ELISA. Th2-high asthma (IgE >100 IU/mL and eosinophils≥140 cells/μL, n=110) was stratified into acute attack (n=48), persistent asthma (n=26), and clinical remission (n=36). Additionally, mouse models across asthma stages were established to measure TSLP levels in BALF, serum, and lung tissue, to validate its predictor value.

**Result:**

Serum TSLP was significantly elevated in acute exacerbation and persistent asthma(P<0.01). Multivariable regression confirmed its independent association with remission (OR=1.009, P=0.023). ROC analysis indicated moderate discriminative capacity of TSLP for remission (AUC=0.59, sensitivity=39.1%, specificity=59.4%). Murine models also showed TSLP levels normalization during remission.

**Conclusion:**

Serum TSLP is independently associated with clinical remission in Th2-high pediatric asthma, though its standalone predictive accuracy is moderate (AUC=0.59). Integration with lung function and IgE may form a composite biomarker panel for remission evaluation. This stratification tool may guide asthma risk stratification and personalized disease management. Longitudinal studies are warranted to validate its prognostic utility.

## 1 Introduction

Childhood asthma, a heterogeneous inflammatory airway disorder, imposes significant healthcare burdens due to divergent clinical trajectories and therapeutic responses (1, 2). Current management paradigms advocate phenotype-specific strategies, integrating clinical features with biomarker-based classification (3). Among these, Type 2-high (Th2-high) asthma is the most well-characterized in both children and adults, driven by IL-4/IL-5/IL-13-mediated eosinophilic inflammation and elevated fractional exhaled nitric oxide (FeNO) (4). The ALLIANCE cohort suggested that Th2-high inflammation in childhood may persist into adulthood, contributing to lifelong airway dysregulation underscoring the importance of prognosis evaluation (5). Th2 biomarkers such as IgE and IL-5 are established predictors of therapeutic response to biologics in adults (6, 7), yet their prognostic utility across asthma stages is limited in the pediatric asthma cohort.

Interestingly, conventional Th2 biomarkers (IL-4, IL-5, IL-13, IgE, TARC, and TSLP) showed no significant differences between asthma and control subjects in our study cohort, suggesting that these markers alone may not sufficiently stratify pediatric asthma phenotypes. This finding also highlights the need for more precise asthma stage classification. Specifically, distinguish asthma cohort among acute exacerbation, persistent asthma, and clinical remission, may better evaluate the prognostic utility of Th2 biomarkers.

Asthma remission represents an ununified concept lacking consensus in both clinical and research settings with limited standardized diagnostic criteria, especially in pediatric populations (8, 9). Previous studies indicate that asthma remission varies considerably in different cohorts and is inconsistently defined by prolonged symptom-free periods without exacerbations or medication use (8, 10, 11). Population-based cohorts indicate that approximately 20% of children achieve sustained remission (≥12 months without symptoms/exacerbations), while a majority progress to persistent disease into adulthood (12–14). Given the increasing emphasis on asthma remission as a pivotal therapeutic objective, defining and predicting remission status becomes a critical unmet clinical need, particularly in pediatric populations (9).

In this context, we hypothesized that serum biomarker levels correlate with clinical remission in pediatric Th2-high asthma patients, potentially serving as predictive markers for asthma remission when integrated with clinical parameters.

We conducted a cross-sectional clinical study coupled with a murine asthma model, mainly comparing baseline clinical characteristics and serum biomarker profiles among Th2-high pediatric asthma patients stratified by asthma stage—acute exacerbation, persistent asthma, and clinical remission. We rigorously defined clinical remission through literature-based criteria: 1) absence of significant asthma symptoms or exacerbations for at least 12 months, 2) consensus among pediatric pulmonologists, 3) and no systemic corticosteroid use during this period (8, 10, 11, 15).

Our baseline analysis revealed that thymic stromal lymphopoietin (TSLP), an epithelial-derived cytokine critical in Th2 inflammation initiation and airway remodeling (16–18), is positively correlated with IL-4, IL-5, IL-13, TARC, and Periostin, reinforcing its central role in Th2-driven inflammation. Furthermore, Th2 high subgroup analyses revealed that serum TSLP level was uniquely associated with the asthma stage. Higher serum TSLP was significantly correlated with persistent asthma and frequent exacerbations, while lower TSLP levels were linked to clinical remission. Importantly, serum TSLP level showed significant correlations with established asthma parameters, including total IgE and key lung function indices (FEV1/FVC and FEF25-75% predicted), with multivariable regression confirming its independent association with remission (OR=1.009, P=0.023). Receiver operating characteristic (ROC) analyses further validated its discriminatory ability for asthma remission, underscoring its potential predictive utility.

To strengthen these findings, we employed a murine model of asthma progression, demonstrating that TSLP levels in lung tissue, BALF, and serum were significantly elevated during the chronic asthma stage but decreased in remission, reinforcing its potential relevance as a biomarker for asthma trajectory assessment.

By integrating TSLP with lung function indices and IgE levels, we propose a composite biomarker panel to predict disease outcomes and guide remission-focused strategies. These findings provide novel insights into the immunological mechanisms underlying progression and remission in pediatric asthma, offering a potential tool for risk stratification in asthma management.

## 2 Methods

### 2.1 Study design and population

This study enrolled 172 children, including 119 asthmatic subjects diagnosed according to the 2022 GINA guidelines and national pediatric asthma guidelines at the Third Affiliated Hospital of Zhengzhou University, and 53 age- and sex-matched healthy controls. Control subjects were recruited from community health programs, ensuring a representative sample. Inclusion criteria for the control group required no history of respiratory symptoms, no need for respiratory medications, normal lung function tests, and no family history of asthma or atopy. Exclusion criteria included a prior diagnosis of asthma, allergic rhinitis, eczema, or any other chronic respiratory disease. To further minimize confounding, children with recent severe respiratory infections requiring hospitalization were excluded.

### 2.2 Asthma stages classification and clinical remission definition

T2-high asthma was defined as IgE >100 IU/mL and blood eosinophils ≥140 cells/μL, according to prior literature and GINA 2022 guidelines (3, 19).

Th2 high asthma subjects (n=110) were further categorized into three groups: acute attack asthma, persistent asthma, and clinical remission.

1) Acute exacerbation (n=48): Defined by physician-diagnosed worsening wheezing, shortness of breath, cough, or chest tightness at the time of enrollment (3, 20).

2) Clinical Remission (n=36): Currently lacks a universally accepted definition.

We adopted a rigorous, literature-based definition of clinical remission, integrating clinical and functional markers to classify our patient cohort. Specifically, they met the following criteria: No significant asthma symptoms or exacerbations for≥ 12 months; Consensus among pediatric pulmonologists on remission status; No use of systemic corticosteroids during the remission period. This classification aligns with the remission framework proposed by Menzies-Gow et al. and Thomas et al., which distinguishes on- and off-treatment clinical remission from complete remission (10, 11).

3) Persistent Asthma (n=26): Presence of asthma symptoms within the last year or daily long-term controller medication use for symptom control (13, 21).

All enrolled asthma patients received guideline-directed standard therapies tailored to their disease severity under pediatric pulmonologists’ supervision, with no use of monoclonal antibody treatments during the study period.

This study was conducted in accordance with the Declaration of Helsinki, International Council for Harmonisation (ICH) guidelines for Good Clinical Practice, and local regulatory requirements. The protocol (2021-108-01) was initially approved by the ethics committee of the Third Affiliated Hospital of Zhengzhou University. All participants and their parents granted their written informed consent and all experiments were performed per the approved guidelines and regulations.

### 2.3 mice

BALB/c mouse, female, SPF grade, weight 18 ± 2g, 6–8 weeks old, were purchased from Shanghai Jiesjie Laboratory Animal Co., LTD. They were fed in an SPF environment with free access to sterilized water and food, controlled with a 12-hour daytime to 12-hour night cycle, indoor temperature of 20-30 °C, and humidity of 50%. After acclimation for 1 week, the mice were randomly divided into 4 groups (control, acute asthma, chronic asthma, and asthma remission), 6 in each group. Mice in the control group were given 0.2mL normal saline intraperitoneal injection, and other asthma model groups were all intraperitoneally injected with 0.2mL suspension which is complexed 20μg of OVA (GradeV) with 2mg Aluminum hydroxide on days 0, 7, and 14 to induce asthma. To mimic different stagings of human asthma, OVA-sensitization mouse asthma models undergoing different durations of OVA-inhaled stimulation. From day 21, mice in the acute asthma group were challenged sensitization by ultrasonic atomizing inhalation of 3% OVA (Grade II) solution in the closed container for 30 minutes each day, lasting for 7 days (22). The same atomization condition applied for the chronic asthma model group was 3 times a week, continuing for 6 weeks (23). The remission group was atomized once a month for 2 months (24). After the ceases of OVA inhalation exposure, all the experimental groups were determined for airway hyperresponsiveness (AHR) using different concentrations of methacholine. Th2 diagnostic inflammation biomarkers were detected including eosinophils, specific-IgE, and Th2 cytokines (IL-4, IL5, and IL-13), both in blood and BALF. An overview of the results showed that the three murine models of asthma were successful. Compared with the control group, the other three groups were found airway methacholine hyperresponsiveness. Higher IgE and Th2 cytokines were detected in serum and BALF, and also more eosinophil infiltration in the lungs. All animal experiments are approved by the Laboratory Animal Ethics Committee (IACUC) of Fudan University and are conducted in strict accordance with the laboratory animal management regulations of Fudan University (2022JS Huashan hospital-497).

### 2.4 Study measurements

#### 2.4.1 Clinical information collection

All subjects’ holistic history including signs and symptoms of asthma, medication use, and common triggers observed by parents are collected by well-trained clinicians. Height and weight were measured and body mass index (BMI) was calculated as an index of the physique.

#### 2.4.2 Lung function tests

Asthma-related pulmonary function was measured according to international standards. The forced expiratory volume in one second (FEV1) and forced vital capacity (FVC) were calculated using the formula of the American Thoracic Society/European Respiratory Society (ATS/ERS) guidelines (25). FEF25-75% pred were also recorded (26).

### 2.5 Inflammatory parameters

The fraction of exhaled nitric oxide was measured at a flow rate of 50 ml/s according to the recommended standards (SV-02, Wuxi Shangwo, China) (27). Differential white blood cell counts were performed using the automatic blood cell count with a full automatic hemocyte analyzer (BC-5800, Mindary, Shenzhen, China). Serum total IgE was measured in an automatic biochemical analyzer (AU5400, Beckman Coulter Inc, Calif, USA) and related reagents were from Diasys diagnostic systems (Diasys, Catalog No: 17239).

### 2.6 Skin tests

Skin prick testing (SPT) for common aeroallergens such as Dermatophagoides pteronyssinus and farina was measured among children. Grass and tree pollen mix was also included, food allergens, molds, and animal dander. Histamine (10 mg/mL of histamine phosphate) and 0.9% saline were used as positive and negative controls, respectively. The positive reaction was a wheal observed 3 mm greater than the negative control (28).

### 2.7 Serum concentrations of biomarkers and cytokines

Venous blood samples were collected with a vacuum blood tube and centrifuged at 3000 g for 10 mins. Then the serum was isolated and stored in aliquots at −80°C until use. Levels of serum IL-5, IL-13, IgE, IL-4, Periostin, TARC, and TSLP were measured by using specific human enzyme-linked immunosorbent assay kits (Shanghai Wellbio Technology Co., Ltd, No: EH6305M, EH6261M, EH6244M, EH6304M, EH6393M, EH6079M, EH6521M), according to the manufacturer’s instructions. Each sample was tested in duplicate. The detection limits of the ELISA assays were as follows: IL-4 (LOD: 1.3 pg/mL, ULOQ: 250 pg/mL), IL-5 (LOD: 4.2 pg/mL, ULOQ: 1000 pg/mL), IL-13 (LOD: 4.6 pg/mL, ULOQ: 1000 pg/mL), IgE (LOD: 0.62 ng/mL, ULOQ: 100 ng/mL), Periostin (LOD: 0.054ng/m1, ULOQ: 10ng/ml), TARC (LOD: 12.5 pg/mL, ULOQ: 2000pg/mL), TSLP(LOD: 13pg/mL, ULOQ: 2000 pg/mL). All biomarker measurements were successfully obtained for all 172 samples (119 asthma patients and 53 controls) with no missing values. No samples had values below the lower limit of detection (LOD) or above the upper limit of quantification (ULOQ) after appropriate dilutions.

### 2.8 Measurement of mouse TSLP by ELISA in serum, BALF, and lung tissue

After the pulmonary function measurement, the mice were exsanguinated by eyeball enucleation. Their sera were separated and stored in aliquots at −80°C until use. Lung lavage fluid was collected using a 1mL syringe that was connected to an indentation needle inserted into the trachea. Next, 0.2mL of pre-cooled PBS solution was injected into the trachea to irrigate the left lung and was quickly withdrawn. This process was repeated three times to ensure proper lavage of the lung. Afterward, the BALF was centrifuged at 4°C for 15 minutes, and the supernatant was collected and frozen in the -80°C refrigerator for subsequent detection. Lung tissue was divided into different sections in need and stored in the -80°C refrigerator for use. The concentration of mouse TSLP in serum, BALF, and lung tissue was tested by indirect ELISA, using a Mouse TSLP ELISA kit (Shanghai Wellbio Technology Co., Ltd, No: EM30546M). All experiment was performed according to the manufacturer’s instructions.

### 2.9 Statistical analysis

**Figure 1:**
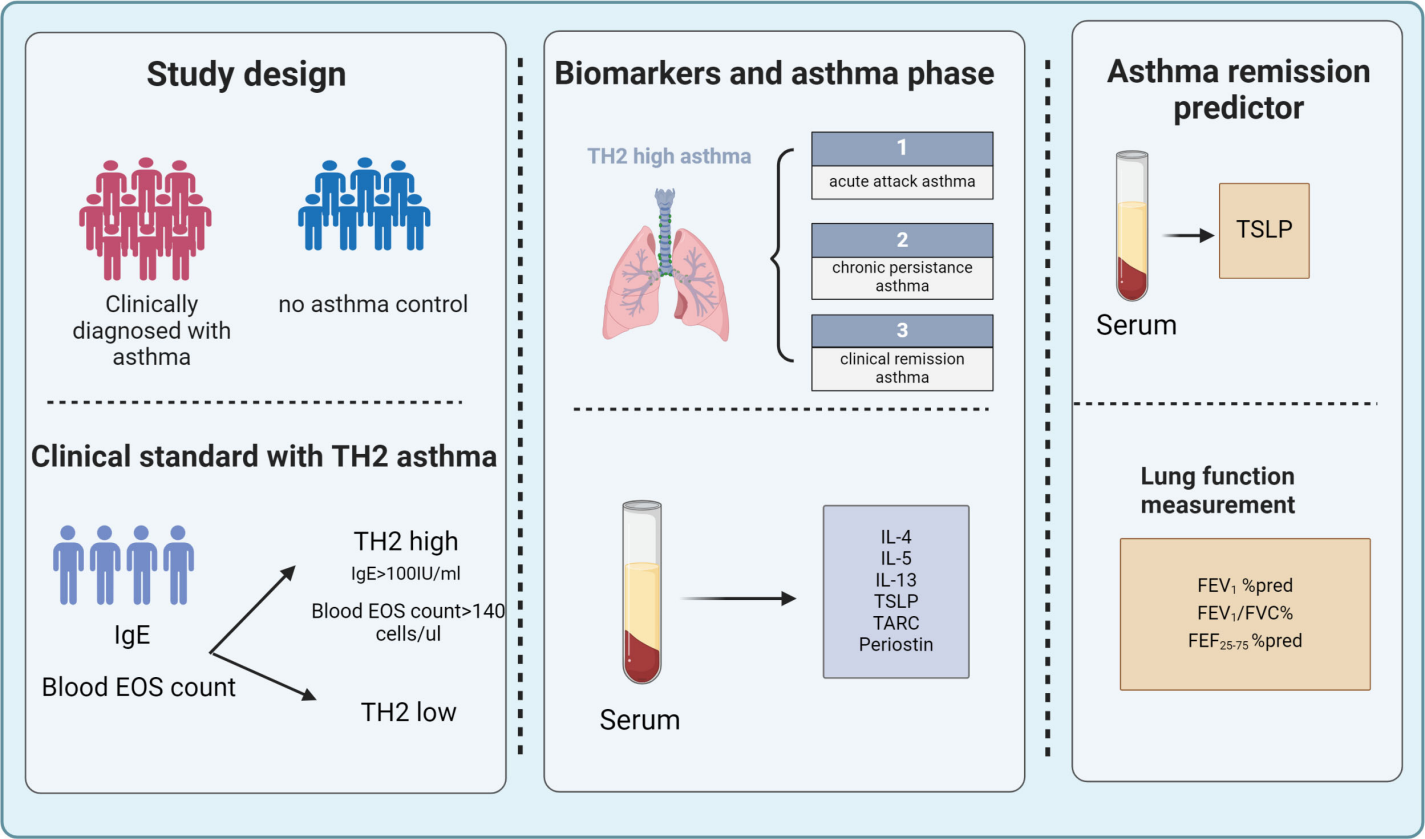
A total of 172 children were enrolled in the study among them, 119 were diagnosed with asthma and 53 were non-asthmatic control subjects. Clinical tests included measurement of lung function, Fraction of exhaled NO (FeNO), total IgE, and blood eosinophil. Serum Th2 biomarkers (TSLP, TARC, IL-4 IL-5, IL-13, IgE, and Periostin) were examined by ELISA. Based on clinical eosinophil count and total IgE, the enrolled patients were readjusted into Th2-high asthma and classified into acute attack asthma, persistent asthma, and clinical remission according to the recent GINA guidelines and clinical evaluation. Serum TSLP levels along with lung function measurements can be used as predictors to help evaluate clinical remission in Th2-high asthma. FeNO, fractional exhaled nitric oxide; IgE, immunoglobulin E; IL, interleukin; TARC, thymus and activation-regulated chemokine; TSLP, thymic stromal lymphopoietin.

Statistical analyses were performed using SPSS v 26.0 (IBM, Chicago, IL, USA) and GraphPad Prism 9 software (GraphPad Software Inc. La Jolla, CA, USA). Normally distributed continuous data were expressed as mean and standard deviation, and non-normally distributed continuous data as median and inter-quartile ranges (IQR). Basic between-group comparisons were made using Mann-Whitney tests, and Student’s t-test, and multiple between-group comparisons were performed using Kruskal-Wallis tests with Dunn’s correction for multiple testing and ANOVA. For categorical variables, the chi-square test or Fisher’s test was conducted. The correlation coefficients among Th2 biomarker levels and clinical characteristics were analyzed using Spearman’s rank correlation coefficient. The association of clinical remission asthma classes with clinical variables was examined using regression models adjusted for potential confounders, including FEV1% pred, FEV1/FVC %, FEF25-75% pred, Total IgE (IU/mL), TSLP (pg/ml). The odds ratio (OR) and 95% confidence interval (CI) were reported. The diagnostic performance of serum TSLP levels to identify predicting biomarkers of clinical remission asthma in children was determined by receiver operating characteristic (ROC) curve analysis. Sensitivity, specificity, positive predictive value (PPV), and negative predictive value (NPV) were calculated for the selected cut-off point. A P level <0.05 was considered statistically significant. A graphic abstract is shown in **Figure 1**.

### 2.10 Language Support

During the manuscript preparation process, ChatGPT (OpenAI, GPT-4, March 2024 version) was used to enhance the readability and linguistic clarity of the text. All AI-assisted modifications were carefully reviewed and verified by the authors to ensure scientific accuracy and integrity.

## 3 Results

### 3.1 Baseline characteristics

In total, 172 children were enrolled, among those 119 were diagnosed with asthma by the clinical physician or still experiencing acute asthma attacks, and 53 were control subjects with no respiratory disease. Baseline demographics, clinical characteristics, and levels of Th2 biomarkers of the overall population are reported in **Table 1**. Children with asthma had significantly higher blood eosinophil counts and blood eosinophils (%) than controls (P<0.05), while there were no significant differences regarding age and sex. As for the Th2 biomarkers(IL-4 P=0.142, IL-5 P=0.4791, IL-13 P=0.6885, Serum IgE P=0.2534, TARC P=0.2074, TSLP P=0.6571), there are no significant statistical differences observed between asthma and control subjects.

**Table 1:** Demographic and laboratory characteristics of the study groups (n=172).

Variables	Asthma n=119	Control n=53	P value
Sex(M/F)	(70/49)	(31/22)	0.967
Age(years)	6.7 ± 3.1	7.2 ± 2.9	0.2048
Eosinophils count/l	0.26 (0.13-1.7)	0.16 (0.1-0.7)	**0.0413**
Blood eosinophils (%)	2.1 (1.3-3.225)	3.35 (1.5-5.75)	**0.014**
IL-4(pg/ml) Mean ± SD Median(min-max)	50.85 ± 22.11 48.56(15.08-175.7)	54.26 ± 17.94 53.85(17.06-107.1)	0.142
IL-5(pg/ml) Mean ± SD Median(min-max)	246.3 ± 114.9 238.8(52.94-890.5)	241.7 ± 102.8 229.8(78.38-597.5)	0.4791
IL-13(pg/ml) Mean ± SD Median(min-max)	174.3 ± 70.92 178.8(50.74-335.8)	165.5 ± 56.48 172.9±(39.75-263.9)	0.6885
Serum IgE(ng/ml) Mean ± SD Median(min-max)	31.93 ± 20.11 27.21(7.345-109.2)	29.32 ± 20.42 22.15(7.832-104.3)	0.2534
Periostin(ng/ml) Mean ± SD Median(min-max)	1.169 ± 0.7573 1.002(0.2556-4.772)	1.275 ± 0.7091 1.193(0.07387-3.106)	0.2074
TARC(pg/ml) Mean ± SD Median(min-max)	251.7 ± 93.31 247.8(54.47-533.5)	265.9 ± 81.19 265.3(48.53-426.8)	0.2159
TSLP(pg/ml) Mean ± SD Median(min-max)	374.2 ± 285.7 297.2(72.38-1400)	356.6 ± 285.1 250.2(86.81-1589)	0.6571

Data are presented as a percentage, median (inter-quartile range), or mean ± standard deviation. Values in bold are statistically significant. IgE, immunoglobulin E; IL, interleukin; SD, standard deviation; TARC, thymus, and activation-regulated chemokine; TSLP, thymic stromal lymphopoietin.

### 3.2 Baseline correlations between biomarkers

**Figure 2:**
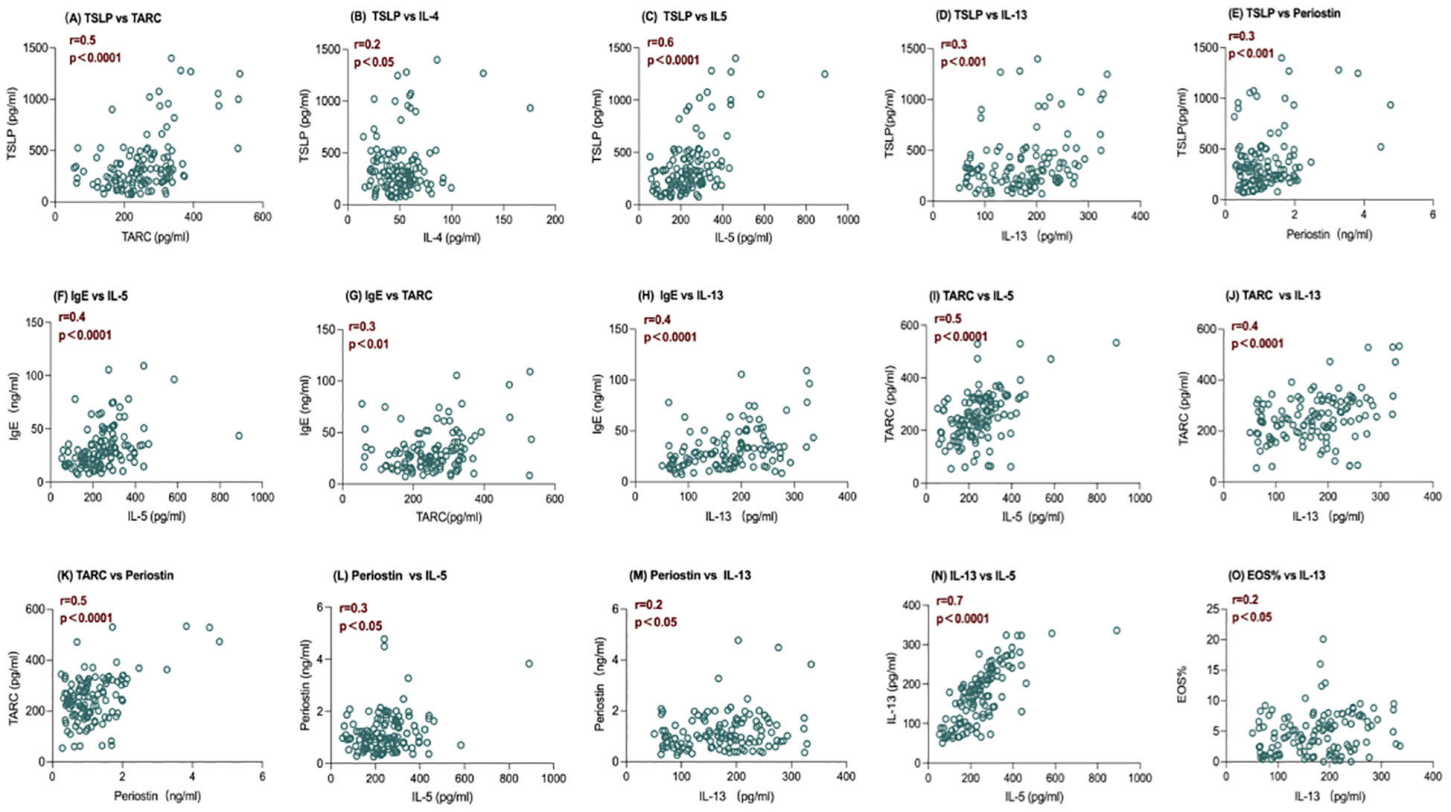
Baseline correlations between serum levels of IL-5 and IL-13 and other biomarkers of inflammation. Spearman correlation coefficients (r) and nominal P value are shown. **(A–E)** The level of serum TSLP has positive correlations with TARC, IL-4, IL-5, IL-13, and Periostin, with P <0.005 in all; **(F–H)** The level of serum IgE has positive correlations with IL-5, TARC, and IL-13,with P <0.0001 in all; **(I-K)** The level of serum TARC has positive correlations withand IL-5, and IL-13 , ,and Periostin with P <0.0001 in all; **(L, M)** The level of serum Periostin has a positive correlation with IL-5 and IL-13 with P <0.05 in all; **(N)** The level of serum IL-13 and IL-5 are positively correlated at baseline with nominal P < 0.0001; **(O)** The level of serum IL-13 was the only Th2 biomarker positively correlated with clinically measured blood eosinophils (%) at baseline with nominal P<0.05. IL, interleukin; TARC, thymus, and activation-regulated chemokine; TSLP, thymic stromal lymphopoietin; Eos%, blood eosinophils (%), n=119.

At baseline, the level of serum TSLP has positive correlations with TARC (r =0.5), IL-4 (r =0.2), IL-5 (r =0.6), IL-13(r=0.3), and Periostin (r = 0.3) (*P <*0.005 in all) (**Figures 2A–E**). Positive correlations were also observed at baseline for IgE with IL-5 (r = 0.4), TARC (r =0.3), and IL-13 (r = 0.4) (*P <*0.0001 in all) (**Figures 2F–H**). There were also positive correlations between TARC and IL-5 (r = 0.5), and IL-13 (r =0.4), as well as between TARC and Periostin (r=0.5) (*P <*0.0001 in all) (**Figures 2I–K**). Periostin had a positive correlation with IL-5 (r =0.3) and IL-13 levels (r=0.2) at baseline(P <0.05 in all) (**Figures 2L, M**). IL-13 and IL-5 (r =0.7) levels were positively correlated at baseline (**Figure 2N**), with nominal *P* < 0.0001. Notably, IL-13 was the only Th2 biomarker positively correlated with clinically measured blood eosinophils (%) at baseline (r =0.2) with nominal *P*<0.05 (**Figure 2O**). No correlations were found between IgE, Periostin, IL-4, and Blood eosinophils (%) with any of the other biomarkers assessed.

### 3.3 Clinical features of type-2-high patients

Th2-high asthma criteria were defined based on blood eosinophil counts and allergen-specific serum IgE: IgE >100 IU/ml and a blood eosinophil count ≥140 cells/μl. The enrolled subjects were recategorized into Th2 high asthma (n=110) and controls (n=53). The Th2 high subjects were ulteriorly grouped into three categories (acute asthma, persistent asthma, and clinical remission), those who presented with worsening wheezing, shortness of breath, cough, and chest tightness, and were diagnosed as acute asthma attacks by physicians were defined as asthma cases in acute exacerbation or acute asthma attack (n=48). Asthma cases in clinical remission (n=36) were characterized by an absence of asthma symptoms and medicine use for at least ≥1 year with no acute asthma attack within a year. Persistent asthma (n=26) was defined as those with the presence of asthma symptoms in the last year or the daily use of long-term control medications to achieve and maintain control in the last year. A comparison of clinical characteristics is presented in **Table 2**. Gender or age was not a factor. No significant differences were found for allergic rhinitis, blood eosinophils (%), total IgE levels, aeroallergen sensitization, and FeNO (parts/billion) between groups. Notably, differences were statistically significant concerning lung function measurements including FEV1% pred (P=0.015), FEV1/FVC % (P=0.40), and FEF25-75% pred (P=0.0021).

**Table 2:** Characteristics of patients defined as acute attack asthma, persistent asthma, and clinical remission.

Variables	Acute Attack Asthma n=48	Persistent Asthma n=26	Clinical Remmision n=36	P value
Sex(M/F)	(33/21)	(13/14)	(24/14)	0.431
Age(years)	6.9 ± 2.7	6.5 ± 3.5	6.6 ± 3.1	0.816
Allergic rhinitis	48.1%	42.1%	51.9%	0.732
FEV1% pred	80.15 ± 18.86	92.90 ± 14.23	103.0 ± 44.65	**0.0015**
FEV1/FVC %	92.77 ± 14.48	99.62 ± 6.573	95.22 ± 22.45	**0.040**
FEF25-75% pred	56.53 ± 24.42	81.44 ± 25.66	71.74 ± 17.38	**0.0021**
FeNO(parts/billion)	20.72 ± 22.76	20.79 ± 24.52	21.57 ± 23.69	0.480
Blood eosinophils (nl)	0.46 (0.06-1.66)	0.36 (0.05-1.13)	0.33 (0.05-0.78)	0.082
Total IgE (IU/ml)	160.9 (5.2-1814)	104 (12.6-1346)	44.8 (8-1905)	0.500
Skin tests				
Dermatophagoides pteronyssinus and farina	21.7	26.3	29.2	0.581
Animal dander	8.7	21.1	8.3	0.674
Food allergen	30.4	31.6	29.2	0.569
Grass,tree and pollen mix	21.7	10.5	8.3	0.347
Molds	4.3	5.3	8.3	0.940
Aeroallergen sensitization	60.9	47.4	50	0.452

Data are mean ± SD, median (interquartile range), or percentage. Values in bold are statistically significant. FEV1, forced expiratory volume during the first second; FVC, forced vital capacity; FEF25-75% pred, forced expiratory flow between 25% and 75%, predicted.

### 3.4 Th2 biomarkers of clinical Th2 high asthma groups

**Figure 3:**
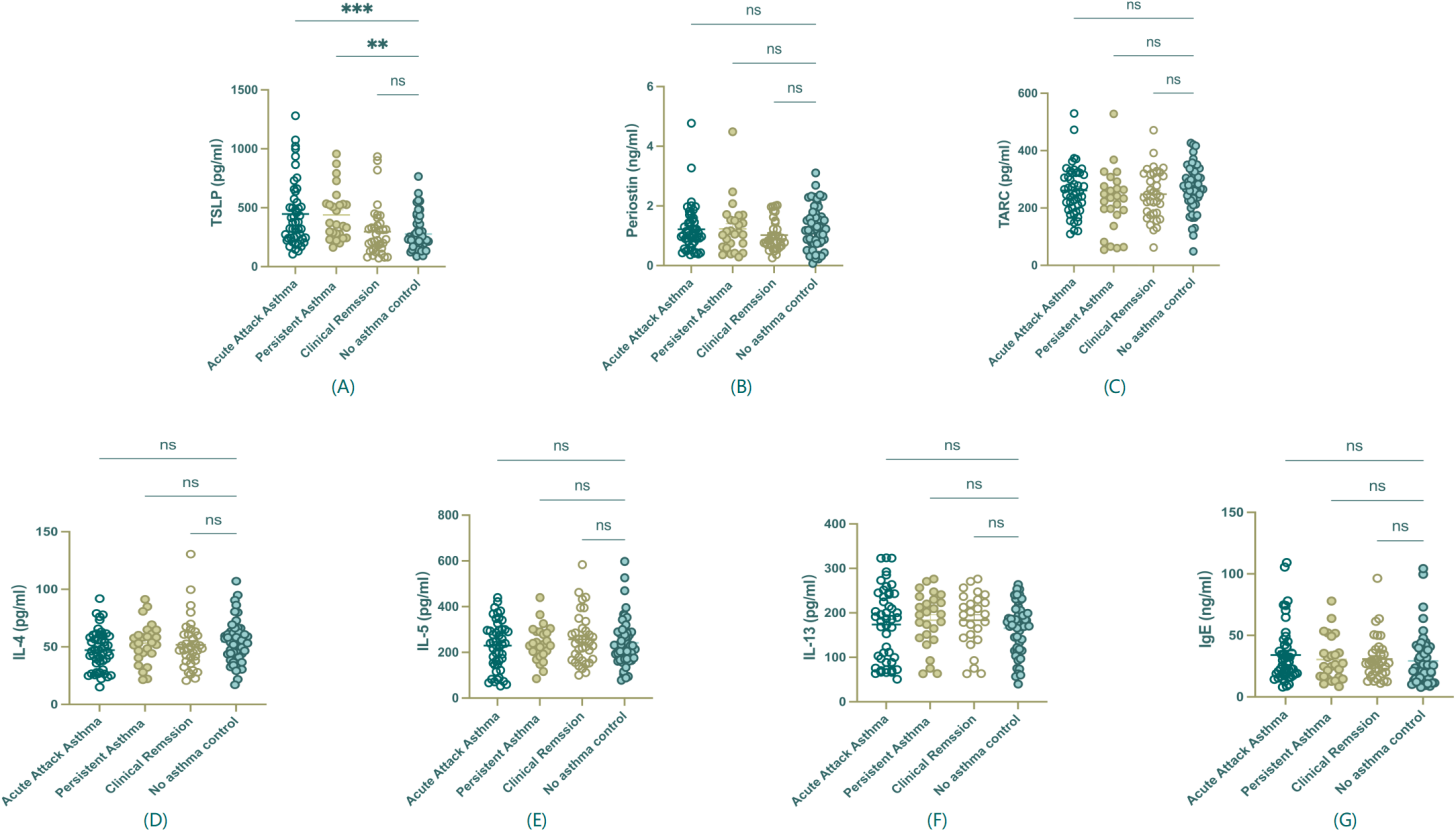
Serum biomarker levels among asthma groups. **(A)** TSLP showed a statistically significant difference among groups. **(B-G)** Periostin, TARC, IL-4, IL-5, IL-13, and IgE, did not show significant differences among groups and are presented for reference purposes only. **P<0.01, ***P < 0.001, ns, non statistical significance.

As shown in **Figure 3**, serum TSLP was the only biomarker that exhibited a statistically significant increase in acute exacerbation and persistent asthma compared to remission and control groups (P<0.01, **Figure 3A**), highlighting its potential prognostic value in pediatric Th2-high asthma. In contrast, other Th2 biomarkers, including Periostin, TARC, IL-4, IL-5, IL-13, and IgE, did not show statistically significant differences among groups (**Figures 3B–G**). These non-significant results are presented for reference and completeness.

### 3.5 Correlation between serum TSLP level and clinical variables in Th2 high asthma

Spearman’s correlation coefficients were conducted to investigate whether serum TSLP level has associations with subjects’ clinical characteristics in Th2-high asthma and whether it can be used as a predictor to evaluate the ongoing eosinophilic inflammation remission. The results are shown in (**Table 3**). Serum TSLP level was found significantly correlated with total IgE (Spearman’s rho [r] = 0.48, P < 0.0001), FEV1/FVC ratio (r = −0.365, P < 0.05), and FEF25-75% pred (r= −0.06, P <0.05), whereas no correlations were found with age, BMI, or allergic rhinitis, atopic dermatitis, blood eosinophil counts.

**Table 3:** Spearman’s correlation coefficients between serum TSLP level and other clinical characteristics in children with Th2 high asthma.

Variables	Serum TSLP(pg/ml)	P value
	r	
Age, y	-0.03	0.770
BMI	-0.081	0.455
Atopic dermatitis	0.372	0.155
Allergic rhinitis	0.098	0.119
Total IgE (IU/mL)	0.482	**<.0001**
Eosinophils (%)	-0.072	0.502
FEV1% pred	-0.106	0.457
FEV1/FVC ratio	-0.365	**0.008**
FEF25-75% pred	-0.306	**0.031**

BMI, body mass index; FEF25-75, forced expiratory flow between 25% and 75%; FEV1, forced expiratory volume during the first second; FVC, forced vital capacity. Value in bold is statistically significant.

### 3.6 Multivariable regression and ROC analyses for predictors of clinical asthma remission and persistence in Th-2 high asthma

To find out which predictors show relationships with clinical remission in Th2-high asthma, a multiple regression model was designed including the variables showing differences between readjusted Th2-high groups and variables associations with TSLP in Spearman’s analyses. No positive result was found in the analysis of multivariable logistic regression in determining the predictors of Th2-high persistent asthma. However, serum TSLP was confirmed as an independent predictor of remission (OR=1.009, 95% CI:1.008–1.008, *P*=0.023), while lung function indices (FEV_1_% pred, FEV_1_/FVC) and IgE showed no significance (**Table 4**).

**Table 4:** Multivariable logistic regression analysis for clinic remission in Th2 high asthma.

Variable	OR (95% CI)	P Value
FEV1% pred	0.963 (0.959-0.966)	0.411
FEV1/FVC ration	1.085 (1.096-1.074)	0.189
FEF25-75% pred	0.974 (0.972-0.976)	0.568
Total IgE (IU/mL)	1.014 (1.014-1.013)	0.668
TSLP (pg/ml)	1.009 (1.008-1.008)	**0.023**

Value in bold is statistically significant at p < 0.05.

**Figure 4:**
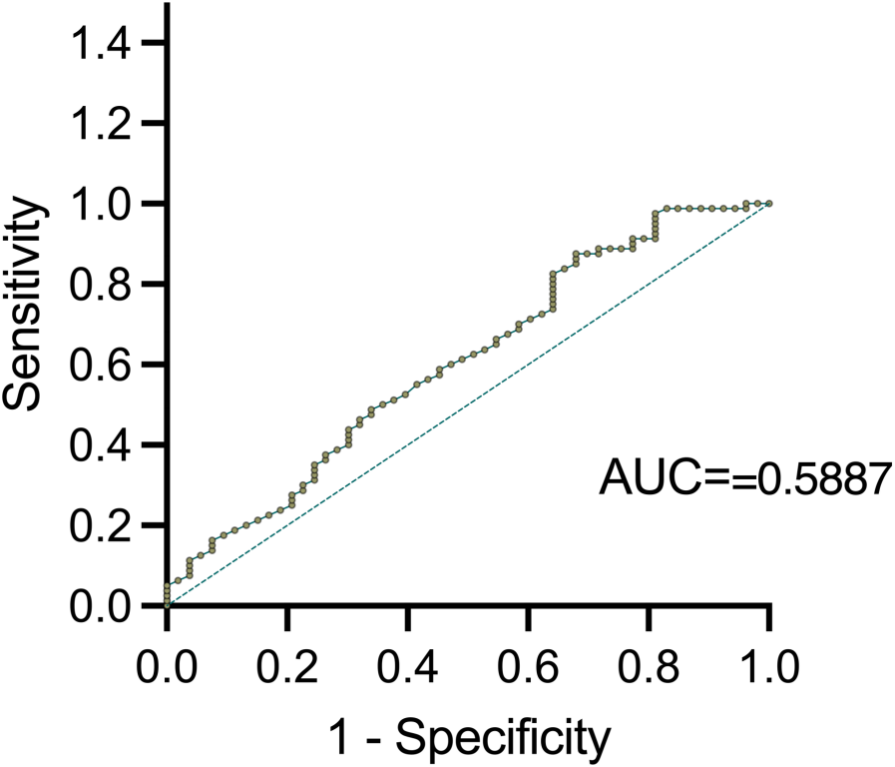
ROC curve for clinical remission in Th2 high asthma.

ROC curves analysis was further conducted to validate serum TSLP level correlated with clinical remission in Th2-high asthma (AUC=0.5887, 95% CI: 0.5052 to 0.7038, *P <*0.05) (**Figure 4**). The optimal TSLP cut-off value of 373.363 pg/ml was found to have the highest clinical sensitivity and specificity. PPV and NPV of 100%, 39.1%, and 59.4%, 100%, respectively.

Since the AUC value of 0.5887 does not exceed the threshold of 0.7 for acceptable discrimination, it indicates that larger sample sizes, additional biomarkers, and longitudinal data need to be included to enhance the predictive model for future studies. Additionally, the optimal TSLP cutoff (≈373 pg/mL) provided only 39.1% sensitivity and 59.4% specificity for predicting remission, underscoring the limited accuracy of TSLP alone.

### 3.7 Comparison of TSLP in serum, BALF, and lung tissue of different mouse model groups

**Figure 5:**
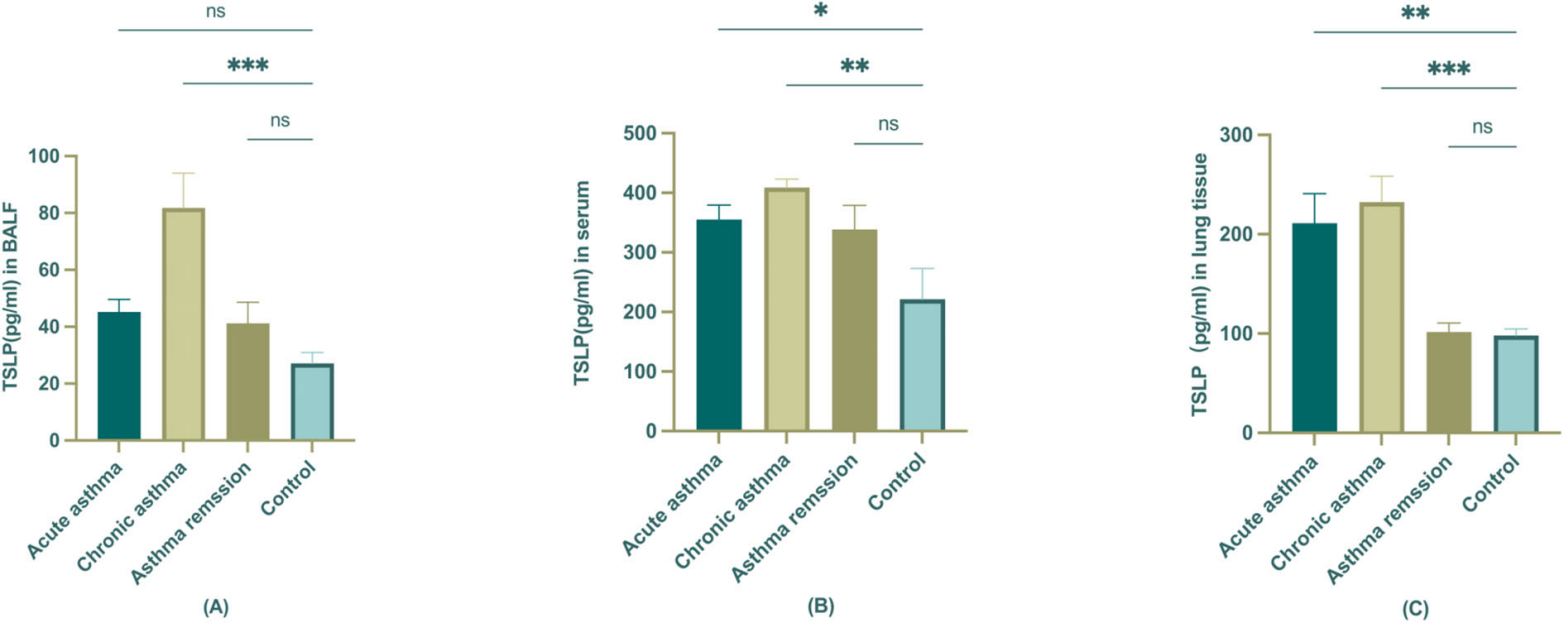
The expression of TSLP in BALF, serum, and lung tissue of different stages of asthmatic mice. **(A)** TSLP level in BALF; **(B)** TSLP level in serum; **(C)** TSLP level in lung tissue. The statistical analysis compared control group is represented as *P<0.05; **P < 0.01; ***P<0.001 (n=6).

The hallmark of the Th2-high asthma was IL4, IL5, and IL13, and the mouse Elisa kit was used to screen the Th2 cytokines in BALF and serum of different stages mouse models first (data not shown). Measurements of TSLP levels were then examined in serum, BALF, and lung tissue of different stages of mouse asthma models to confirm and validate the observations from the study population. We aimed to inspect the expression of TSLP in different tissues of asthmatic mice and to find out the most indicative significance. When comparing the concentration of TSLP in serum, BALF, and lung tissue, it was found that BALF had the lowest level of TSLP concentration. There was no statistically significant change in the acute and remission stages when compared to the control, but TSLP did increase noticeably in the chronic stage (**Figure 5A**). There seems to be no significant difference in the levels of TSLP in mouse serum between control and asthma remission. However, it had been noted that during the acute and chronic stages, TSLP levels increased, as depicted in the figure. (**Figure 5B**) Furthermore, the lung tissue also demonstrated a comparable trend as serum, with significant statistical significance observed in this case (**Figure 5C**).

## 4 Discussion

This study demonstrates that serum TSLP levels are significantly associated with clinical asthma remission, providing a potential biomarker for disease monitoring. In addition, it extended previous investigations regarding identifying the potential biomarkers to predict clinical remission in Th2-high pediatric asthma. We found that when subjects were simply partitioned into asthma and control, no significance was observed in Th2 biomarkers between these two groups, whereas clinical measurements including blood eosinophils (%) and eosinophils count were statistically significant. When we defined Th2 high asthma based on eosinophils count (≥140 cells/μl) and total IgE (IgE >100 IU/ml), the serum TSLP levels were observed higher in subjects experiencing acute exacerbation and persistent asthma compared with no asthma control subjects. It correlated with key lung function parameters, including the FEV1/FVC ratio, FEF25-75% predicted, and total IgE levels. In addition, our results also found an association between serum TSLP levels and clinical remission independent of confounding factors such as lung function measurements or allergy sensitization. To further validate these findings, we employed a mouse model of asthma progression, demonstrating TSLP upregulation in chronic asthma and attenuation during remission. These results position TSLP as a novel biomarker for clinical asthma remission when combined with IgE levels and lung function metrics, offering mechanistic insights into Th2 inflammation resolution in pediatric asthma.

### 4.1 Th2 Biomarkers and clinical relevance

Asthma is a heterogeneous disease, and the transition from phenotype-based classification to endotype-driven stratification has refined our understanding of its pathophysiology (1). Phenotypes are typically defined by clinical characteristics, allergen sensitization patterns, and lung function measurements, whereas endotypes are classified based on underlying molecular and immune mechanisms (29). Thus, there is a growing urge to investigate asthma by connecting discernible and stable biomarkers with clinical characteristics to predict asthma outcomes (30).

Th2-high asthma, one of the most well-characterized endotypes, is driven by IL-4, IL-5, and IL-13-mediated eosinophilic inflammation, airway remodeling, and epithelial dysfunction (7, 31). Th2-high asthma accounts for approximately 50% of asthma cases, but its identification remains challenging due to variability in biomarker expression (1, 32). Our findings align with this complexity, there were no significant differences in baseline Th2 biomarker levels, suggesting that lumping all asthma cases together may obscure subtype-specific biomarker trends, particularly when the study population was not recruited specifically with Th2-high characteristics. To address this heterogeneity, we stratified patients into Th2-high and Th2-low subgroups. Among the 119 asthma patients, 9 were categorized as Th2-low. This finding of a small Th2-low subgroup supports the notion that our initial comparison may have been confounded by the inclusion of patients with varying asthma phenotypes and may have affected the power to detect significant differences in biomarker levels at baseline.

Interestingly, when investigating the correlations between the biomarkers and clinical characteristics, only IL-13 showed a significant positive association with blood eosinophils (%), while other Th2 biomarkers did not correlate with eosinophil counts or IgE levels. This contrasts with findings from Corren, Pham, et al., who found that baseline levels of serum IL-5, IL-13, and Periostin correlated significantly with baseline blood eosinophil counts and FeNO levels in uncontrolled severe asthma subjects. The differences in disease severity may account for such results (33, 34).

### 4.2 Asthma remission, persistence and predictive role of Th2 biomarkers

Asthma remission is increasingly recognized as a key therapeutic goal, yet it lacks a standardized definition (9). Several large-scale longitudinal studies have tracked asthma remission in both pediatric and adult populations. Almqvist et al. (35),conducted a 15-year follow-up study and found that adult-onset asthma rarely achieves remission, with most patients requiring long-term controller therapy. However, early-onset asthma, particularly in childhood, appears to have a higher likelihood of remission, underscoring the importance of early intervention (13).

With the increasing use of biologics for asthma treatment and the shift from phenotype-based to endotype-driven classifications, integrating key clinical predictors with the biomarker-driven framework may improve prognostic accuracy in pediatric asthma, especially regarding the role of Th2 biomarkers in remission prediction (36). Previous studies have emphasized the role of Th2-associated biomarkers in allergic asthma, particularly in individuals with grass pollen sensitivity (37, 38). Building on these insights, we further stratified our study population into three groups: acute asthma exacerbation, persistent asthma, and clinical remission to investigate the relevance of Th2 biomarkers across different childhood asthma stages. As described in the Methods section, Th2-high asthma was defined based on specific criteria, ensuring accurate subgroup and classification. Our findings revealed that lung function indices, including FEV1%, predicted, FEV1/FVC ratio, and FEF25-75% predicted, were significantly different across asthma groups, supporting prior research indicating that lung function parameters are highly sensitive markers for asthma remission (13, 39, 40). When analyzing circulating levels of Th2 biomarkers across asthma groups, serum TSLP was the only biomarker that exhibited significant differences, with elevated levels in acute exacerbation and persistent asthma compared to remission. Notably, research has demonstrated that allergic rhinitis (AR) and other atopic conditions share Th2 inflammatory pathways with asthma (41), indicating that TSLP can be induced by nasal epithelial stimulation upon allergen exposure. In our cohort, approximately half of the asthmatic children had concurrent AR (48–52% across subgroups, **Table 2**), but AR prevalence was evenly distributed among acute, persistent, and remission groups (P = 0.732), minimizing differential bias in our TSLP comparisons. However, despite not introducing systematic bias, AR likely contributed to increased background variability in TSLP measurements. To better delineate the asthma-specific contributions of TSLP. Future studies should stratify or adjust for such co-morbidities to isolate the asthma-specific contribution to serum TSLP.

In contrast, classical Th2 cytokines, including IL-4, IL-5, IL-13, and IgE, did not demonstrate significant variation among groups. However, previous studies have identified potential prognostic roles for these cytokines. For instance, Tan, D.J. et al. found that serum IL-4 and IL-5 levels were predictive of spontaneous asthma remission in adults (42). Additionally, Periostin has been associated with persistent and severe asthma (43, 44), while IL-13 has been recognized as a key regulator of asthma chronicity in murine models (45). TARC (CCL17), a chemokine expressed in Th2 cells, has been proposed as a biomarker for bronchial asthma (46). Furthermore, dynamic changes in plasma TARC concentrations have been observed in children with acute asthma exacerbations, suggesting its potential utility as a disease monitoring marker (47). The variability observed in Th2 biomarker profiles across studies likely reflects the multifractal nature of asthma. Recent genetic studies have identified Gasdermin B (GSDMB) polymorphisms as contributors to enhanced Type-2 cytokine responses in asthma (48), underscoring potential upstream regulatory mechanisms influencing Th2 biomarkers expression and asthma remission. Additionally, differences in patient populations, disease severity, and study designs may account for inconsistencies in biomarker findings.

### 4.3 TSLP as a prognostic biomarker for asthma remission: insights from clinical and murine models

TSLP is a key epithelial-derived cytokine of the IL-2 family. With the advancements in TSLP-targeted biologics, it has been widely recognized as a therapeutic target in severe asthma due to its role in driving Th2-mediated inflammation and airway remodeling (49–52). However, whether TSLP can be used as a prognostic biomarker for predicting clinical remission of Th2-high pediatric asthma remains largely unexplored.

Our findings reveal that serum TSLP levels significantly differ across asthma stages, with the highest levels in acute exacerbation and persistent asthma, and the lowest in clinical remission. Furthermore, multivariate logistic regression analysis confirmed TSLP’s independent association with asthma remission (OR=1.009, *P*=0.023), adjusting for confounders. This suggests that TSLP could serve as an indicator in biomarker level to predict remission in pediatric Th2-high asthma. Interestingly, a prior study analyzing sputum Th2 markers found higher baseline TSLP levels in the remission group, and it was not identified as a predictor by univariate regression analysis (6). This discrepancy may be attributed to differences in sample type, study population characteristics, and methodological approaches, highlighting the need for further validation of TSLP as a prognostic biomarker for asthma remission across diverse cohorts. In contrast to prior studies, which focused primarily on TSLP as a therapeutic target rather than a predictive biomarker, our study provides new insights into TSLP’s potential role in immune resolution and disease trajectory in pediatric asthma.

Despite its association with asthma remission, our ROC analysis (AUC = 0.5887) indicates that TSLP alone lacks sufficient predictive power. The relatively small effect size (OR=1.009, *P*=0.023) in our multivariate model further underscores the complexity of asthma remission and the need for multi-biomarker approaches. In support of this, prior studies have demonstrated the synergistic role of TSLP alongside other Th2 inflammation markers, such as Periostin and blood eosinophil counts, in stratifying asthma severity and predicting treatment response (11, 13, 36, 53). Additionally, a multi-center prospective study found that serum TSLP and tryptase levels, combined with blood eosinophil counts, help predict asthma exacerbation risk in severe asthma patients (54).

Additionally, Our baseline analysis showed that serum TSLP exhibited significant correlations with multiple Th2 biomarkers (e.g., IL-5, IL-13, TARC), and the correlation strengths varied, ranging from moderate (r = 0.5-0.6) to weak (r = 0.2-0.3). This moderate correlation likely reflects that multiple inflammatory pathways (Th1, Th17, and non-Th2 mechanisms) contribute to disease progression beyond Th2 dominance (55). Cytokine expression exhibits biological variability influenced by genetic predisposition, environmental exposures, and temporal fluctuations, which may weaken observed associations (56). Given all these complexities, integrating TSLP with other indicators into composite biomarker panels, alongside leveraging longitudinal data and machine-learning approaches, may enhance biomarker-based prediction models and facilitate their translation into clinical practice (57–59).

To further substantiate our clinical observations, we employed a murine model to assess TSLP expression across different disease stages (acute, chronic, and remission). Consistently, TSLP levels were markedly elevated in chronic asthma and declined during remission, detected in lung tissue, BALF, and serum. These results parallel our findings in pediatric asthma, where lower serum TSLP levels were associated with clinical remission. Previous murine studies have demonstrated that TSLP is a key regulator of asthma pathogenesis, with TSLP blockade effectively reducing airway hyperresponsiveness (AHR) and Th2 inflammation (60–62), which further supportsTSLP’s dual function as both a therapeutic target and a potential biomarker for monitoring disease resolution. This convergence of murine and human data highlights the potential of TSLP as both a therapeutic target and a biomarker for monitoring asthma remission.

### 4.4 Conclusion

We report for the first time that serum TSLP can be used as one of the biomarkers to evaluate the remission of asthma, along with other measurements such as lung function and IgE to improve the accuracy. We also testify our findings in murine models. Our findings will be critical for advancing our understanding of biomarker profiles and their clinical relevance in asthma management. We also have highlighted the need for further studies with larger longitudinal studies including Th2-low asthma cohorts to validate the prognostic utility of TSLP and refine its role in biomarker-guided risk stratification. To further explore the biomarker differences of asthma stages.

### 4.5 Limitations and strengths

While our study provides novel insights into the role of TSLP in asthma remission, we acknowledge several limitations that warrant further investigation. First, this is an observational study and does not establish causality but provides critical exploratory data. Second, asthma endotypes are not dominated by Th2 inflammation other endotypes such as Th2-low, neutrophilic, and Th17-high asthma, likely have different biomarker profiles and TSLP-driven pathways. Our study specifically targeted Th2-high asthma, so the results may not directly apply to patients. We emphasize the need for longitudinal validation of our findings in larger cohorts to confirm the stability and reproducibility of TSLP as a remission biomarker. Additionally, comorbid allergic conditions likely contributed to elevated baseline TSLP levels further complicating its role as an asthma-specific biomarker. Given that biomarker research in asthma remission is still in its early stages, our study serves as a stepping stone toward developing validated remission biomarkers, rather than claiming definitive predictive value at this stage. Our findings contribute to this evolving field by proposing TSLP as a potential candidate for future validation, particularly in pediatric populations. Further studies integrating multi-omics approaches and longitudinal patient follow-up will be essential to refine our current understanding and validate these findings for clinical application.

## Data Availability

The raw data supporting the conclusions of this article will be made available by the authors, without undue reservation.
